# Intestinal carriage of invasive non-typhoidal *Salmonella* among household members of children with *Salmonella* bloodstream infection, Kisangani, DR Congo

**DOI:** 10.3389/fmicb.2023.1241961

**Published:** 2023-10-12

**Authors:** Dadi Falay, Liselotte Hardy, Edmonde Bonebe, Wesley Mattheus, Dauly Ngbonda, Octavie Lunguya, Jan Jacobs

**Affiliations:** ^1^Department of Pediatrics, University Hospital of Kisangani, Kisangani, Democratic Republic of Congo; ^2^Department of Clinical Sciences, Institute of Tropical Medicine, Antwerp, Belgium; ^3^Department of Microbiology, Immunology and Transplantation, KU Leuven, Leuven, Belgium; ^4^Department of Microbiology, National Institute for Biomedical Research, Kinshasa, Democratic Republic of Congo; ^5^Division of Human Bacterial Diseases, Sciensano, Uccle, Belgium; ^6^Department of Microbiology, University Teaching Hospital of Kinshasa, Kinshasa, Democratic Republic of Congo

**Keywords:** invasive non-typhoidal *Salmonella*, *Salmonella* carriage, sub-Saharan Africa, *Salmonella* bloodstream infection, households

## Abstract

**Introduction:**

Invasive non-typhoidal *Salmonella* (iNTS), mainly *Salmonella* Typhimurium and *Salmonella* Enteritidis, causes a severe burden in sub-Saharan Africa; however, its reservoir (animal or environmental) is unclear. The present study assessed healthy household members of index patients for intestinal carriage of *Salmonella*.

**Methods:**

Index patients were admitted to the University Hospital of Kisangani (DR Congo), and *Salmonella* was grown from blood cultures. Household members were asked to provide three stool samples for culture for *Salmonella*. *Salmonella* Typhimurium and *S*. Enteritidis isolates from index patients, and household members were assessed for genetic relatedness using the multiple-locus variable number of tandem repeat analysis (MLVA), and the multilocus sequence type (ST) was determined by whole genome sequencing.

**Results:**

Between May 2016 and January 2020, 22 households were visited. The index patient serotypes were Typhimurium, Enteritidis, Typhi, and Paratyphi C; II:42:r:-; and I:7:y:- (*n* = 8, 7, 5, and each 1, respectively). The median (range) delay between the index patient and household sampling was 25 days (2 days to 7.3 months); 203 household members provided at least one stool sample. In all, 15 (7.3%) *Salmonella* carriers were found in nine of 22 households. For one index patient, the household comprised *S*. Typhimurium in four household members, including the index patient, sampled 27 days after bloodstream infection; the MLVA types of these five isolates were similar. They belonged to ST313 lineage 2 and were closely related [0–1 allelic distance (AD) among the stool isolates and eight AD with the blood culture isolate]. In another household, the stool culture of the index patient (obtained 67 days after bloodstream infection) grew *S*. Enteritidis of the same MLVA type; both isolates belonged to the ST11 Central/Eastern African clade and were closely related (three AD).

**Discussion:**

The present study provides evidence of household clustering of *S*. Typhimurium ST313 and intestinal carriage of iNTS several weeks after bloodstream infection.

## Background

Infections caused by non-typhoidal *Salmonella* spp. occur worldwide. In high-income countries, non-typhoidal *Salmonella* serotypes cause self-limiting enterocolitis, but in low-resource settings, they cause bloodstream infections and are currently labeled as “invasive non-typhoidal *Salmonella*” (iNTS) (Feasey et al., 2012; Stanaway et al., 2019). iNTS accounts for ~535,000 invasive bloodstream infections per year, mostly occurring in children under 5 years of age, and is confined to sub-Saharan Africa, where it has a case fatality rate of 17.1% (Stanaway et al., 2019; Marchello et al., 2022). In addition to young age, host risk factors include *Plasmodium falciparum* infection (severe as well as chronic), malnutrition, and anemia (Feasey et al., 2012; Crump et al., 2015; Stanaway et al., 2019). Over 90% of iNTS infections are caused by particular clades of the *Salmonella enterica* subspecies *enterica* serovars Typhimurium and Enteritidis, such as multilocus sequence type (ST) ST313 lineage 1 and multidrug-resistant lineage 2 and ST11 Central/East African and West African clades, respectively (Feasey et al., 2016; Van Puyvelde et al., 2019; Pulford et al., 2021).

In addition to their evolution toward invasive infections, these clades are genetically adapted to human hosts (Feasey et al., 2016; Van Puyvelde et al., 2019; Pulford et al., 2021). This suggests a more restricted host specificity and, as is the case for *Salmonella* Typhi, healthy human carriers as reservoirs for iNTS. In contrast, non-typhoidal *Salmonella* causes enterocolitis and has a broad zoonotic reservoir (Sirinavin et al., 1999; Feasey et al., 2015). Evidence supporting the hypothesis of a human reservoir for iNTS has been provided by studies that compared iNTS isolates from infected patients to *Salmonella* isolates obtained from stool cultures of humans and livestock or other environmental samples close to the infected index patients. Using pulsed-field gel electrophoresis, these studies showed genetic relatedness between the index iNTS isolates and *Salmonella* isolates obtained from stool cultures of healthy humans, whereas they were unrelated to animal or environmental isolates (Kariuki et al., 2002, 2006; Dione et al., 2011; Dekker et al., 2018).

Molecular tools, such as multilocus variable number tandem repeat analysis (MLVA) and whole genome sequencing (WGS), have provided new and more powerful tools for assessing the genetic relatedness of *Salmonella* isolates. Therefore, we conceived an index patient-household study design to provide additional evidence of the human reservoir of the iNTS. Although not conceived as a dual-site study, the design was applied to two iNTS-endemic settings: rural Burkina Faso (Post et al., 2019) and Kisangani, as described in this manuscript. The methods were similar, except that only a single stool sample was sampled in the Burkina Faso study, and livestock and household water were assessed.

The primary objectives of this study were (i) to assess the proportion (frequency) and serotype distribution of *Salmonella* intestinal carriers among household members of index patients with iNTS bloodstream infection; (ii) to assess the genetic relatedness of *S*. Typhimurium and *S*. Enteritidis stool and blood culture isolates; and (iii) to determine the iNTS ST of the *S*. Typhimurium and *S*. Enteritidis isolates. The secondary objective was to assess the antimicrobial resistance profiles of the index patient and household *Salmonella* isolates.

## Methods

### Study site, microbiological surveillance, and index patient blood culture isolates

Kisangani is the capital city of Tshopo Province, located in the northeast of the Democratic Republic of the Congo (DR Congo), with ~1.3 million inhabitants in 2021 [Central Intelligence Agency (CIA), 2023]. Most inhabitants live below the poverty line (data Gouvernement de la Province Orientale, RD Congo). It has a tropical rainforest climate, and *P. falciparum* malaria is holoendemic with perennial transmission (Falay et al., 2016). Similar to other provinces in DR Congo (Tack et al., 2021), iNTS is endemic in Tshopo Province, and recently, a *P. falciparum* outbreak complicated by iNTS infection occurred (Falay et al., 2016).

Since 2008, the University Hospital of Kisangani (UNIKIS) has participated in a national bloodstream infection surveillance network organized by the National Institute for Biomedical Research (INRB, Kinshasa) in collaboration with the Institute of Tropical Medicine (ITM, Antwerp, Belgium). This network provides a free blood culture service integrated into patient care. The purpose of this network is to monitor bacteria involved in bloodstream infections and their antibiotic resistance profiles (Falay et al., 2016, 2022). For the indications, sampling, and workup [identification and antibiotic susceptibility testing (AST)] of blood cultures, we refer to the article by Tack et al. (2021). As part of the surveillance study, blood culture isolates were shipped to the INRB and ITM for reference testing (confirmation, serotyping, and AST) and stored at −80°C for further analysis (Falay et al., 2016, 2022).

### Study design, index patients, and study period

The field study assessed the fecal carriage of iNTS among household members of patients with culture-confirmed iNTS bloodstream infections (index patients). At UNIKIS, children (28 days to 15 years) with *Salmonella* growing from blood cultures were selected as index patients. The head of the household to which the index patient belonged was contacted for recruitment in the *Salmonella* carrier study, which consisted of collecting and culture of *Salmonella* from stool samples for three consecutive days. The study was conducted from March 2016 to March 2020, when it was stopped because of the COVID-19 lockdown decreed in DR Congo.

*Salmonella* Typhimurium and *S*. Enteritidis isolates from blood and stool cultures were compared for genetic relatedness by MLVA. Isolates from households with index patients (household members with matching MLVA types) were assessed using WGS to assess their ST and genetic relatedness expressed in allelic differences (AD). All *Salmonella* isolates were tested for antibiotic susceptibility.

### Household visits, stool sampling, and transport

Upon confirmation of *S. enterica* from blood cultures, the index patient's household address was located. The investigation team visited the household, contacted the household head to explain the study, and invited the household to participate. After obtaining consent, a list of household members was created, and a sampling date was agreed upon. The investigator visited the households the day before sampling. He/she provided a polystyrene container identified by the name, sex, and age of each household member and explained how to collect the stool sample, preferring a morning stool sample. The following morning, the investigator collected the samples between 6 and 8 a.m. and transported them in a cool box to the UNIKIS microbiology laboratory. He/she also provided a container to household members for the next day's sample. The procedure was repeated daily.

### *Salmonella* stool culture

Laboratory processing for stool samples was performed as described previously (Mbuyi-Kalonji et al., 2020). After the reception of samples at the laboratory, ~1 g of each stool sample was suspended in 10 ml of selenite broth (BD Difco, Becton Dickinson and Company, Franklin Lakes, New Jersey) and incubated at 35°C for 12–18 h. Thereafter, 10 μl was inoculated on two *Salmonella-Shigella* (SS) agar plates (Lab M Limited, Lancashire, UK) and incubated at 35°C for 18–24 h and read afterward. In case of no growth, the plates were evaluated after another 18–24 h of incubation at 35°C. In case of growth, up to five colonies suspected to be *Salmonella* were transferred to Kligler Iron Agar (KIA) tubes (Lab M Limited) and incubated for 18–24 h at 35°C. Bacteria grown in the KIA tube and displaying a profile suggestive of *Salmonella* were biochemically confirmed by a panel of disk-based biochemical tests (DiaTabs, Rosco, Taastrup, Denmark). Isolates with a reaction pattern compatible with *Salmonella* were stored in 2 ml tubes of Trypticase Soy Agar (Oxoid, Basingstoke, UK) and shipped to the Institute of Tropical Medicine (ITM, Antwerp, Belgium) for serotyping and AST.

### *Salmonella* serotyping and AST of blood and stool culture isolates

Serotyping of blood and stool culture isolates was performed using commercial antisera (Vison, Pro-Lab Diagnostics Inc., Richmond Hill, Ontario, Canada). AST was done by disk diffusion (Neo-Sensitabs, Rosco, Taastrup, Denmark) and, in the case of azithromycin and ciprofloxacin, by the ETEST macro-method (bioMérieux, Marcy Étoile, France) to assess the minimal inhibitory concentration values (MIC-values) (Tack et al., 2020a). Results were interpreted according to the Clinical and Laboratory Standards Institute (CLSI) M100-S31 criteria (Clinical and Laboratory Standards Institute, 2021). Multidrug resistance (MDR) was defined as combined resistance to amoxicillin, trimethoprim-sulfamethoxazole, and chloramphenicol (Tack et al., 2020b).

### Genetic relatedness between *Salmonella* isolates from index cases and household members

Genetic relatedness between *S*. Typhimurium and *S*. Enteritidis isolates was determined at Sciensano (Brussels, Belgium) by MLVA, as previously described (Falay et al., 2016, 2022). For *S*. Typhimurium, profiles were attributed based on the number of tandem repeats at five loci (STTR9-, STTR5-, STTR6-, STTR10-, and STTR3-). For *S*. Enteritidis, these loci were SENTR7-, SENTR5-, SENTR6-, SENTR4-, and SE3-). Identical MLVA clusters for *S*. Typhimurium were defined as isolates with MLVA types with no or one variation in the rapidly changing loci (STTR5, STTR6, and STTR10) but no variation in the stable loci (STTR3 and STTR9) (Dimovski et al., 2014). For *S*. Enteritidis, a cluster was defined as isolates with variation in none or one of the five loci (Bertrand et al., 2015).

### Whole genome sequencing

*Salmonella* Typhimurium and *S*. Enteritidis isolates from the index patient-household member MLVA clusters were selected for WGS. WGS, including DNA extraction, purification, library preparation, and sequencing (Illumina, San Diego, CA, USA), was performed by Eurofins Genomics (Konstanz, Germany), generating 150 bp paired-end reads. Short reads were assembled *de novo* using SPAdes version 3.6.0.23. Tools integrated into EnteroBase^1^ were used (Falay et al., 2022). Multilocus sequence typing (MLST) was performed using the 7-gene MLST scheme based on the sequences of seven housekeeping genes: *aroC, dnaN, hemD, hisD, purE, sucA*, and *thrA* (Kidgell et al., 2002; Achtman et al., 2012). Hierarchical clustering of cgMLST (HierCC) was performed based on 3,002 locus sequences for *Salmonella* (Zhou et al., 2021; Falay et al., 2022).

### Ethical issues

The study was approved by the ethics committee of the Public Health School of Kinshasa (Comité d'Éthique de l'École de Santé Publique de Université de Kinshasa, ESP/CE/002/2017) and the Institutional Review Board of the ITM (IRB/AB/ac/038, March, 02 2016). Written informed consent was obtained from the heads of households. Oral consent was obtained from all household members. An independent witness was present in cases of illiteracy.

### Data and definitions

For the definition of *Salmonella* index patients and MLVA clusters, refer to earlier paragraphs. A *Salmonella* carrier was defined as a household member who had *Salmonella* growth in at least one of the three stool samples. *Salmonella* isolates with identical serotypes obtained from index patients and corresponding household members were defined as matching isolates or matching pairs. For *S*. Typhimurium and *S*. Enteritidis carriers, an identical or similar MLVA profile was added as a criterion. Household *Salmonella* clusters were defined as ≥2 carriers living in the same household and for whom the same *Salmonella* serotype was isolated from at least one stool sample.

## Results

### Demographic information and serotype distribution of the index patients

During the study period, 43 patients with *Salmonella*-confirmed blood cultures (index patients) were obtained (Table 1). Of these, 22 (51.2%) were obtained from household visits. The reasons for exclusion were unclear addresses and refusal to participate. The median age (range) of the included index patients was 19 months (5 months to 14 years); the oldest children were infected with *Salmonella* Typhi, and 14 (63.6%) were male children. Involved *Salmonella* serotypes were Typhimurium (*n* = 8), Enteritidis (*n* = 7), Typhi (*n* = 4), Paratyphi C, II:42:r:-, and I:7:y:- (one isolate each). Compared to the entire group of index patients, male children were overrepresented, and *S*. Typhimurium infection was slightly underrepresented (eight of 19 index patients; Table 1).

**Table 1:** Serotype distribution and demographic data of patients with blood culture confirmed *Salmonella* infection (index patients).

	**All index patients (*n* = 43)**	**Index patients for whom households were visited (*n* = 22)**
*Salmonella* Typhimurium	19	8
*Salmonella* Enteritidis	13	7
*Salmonella* Typhi	8	4
*Salmonella* Paratyphi C	1	1
*Salmonella* II:42:r:-	1	1
*Salmonella* I:7:y:-	1	1
M/F ratio	1.2	1.75
Median (range) age	30 months (3 days to 27 years)	19 months (5 months to 14 years)

### Household member sampling

The 22 included households (Table 2) comprised 243 household members, of whom 203 (83.5%) committed to participate and provided the first stool sample; 163 and 81 participants (80.3% and 39.9% of those providing the first sample, respectively) provided a second and third stool sample. The median (range) delay between the blood culture sampling of the index patient and the date of the first stool sampling in the corresponding household was 25 days (range, 2 days to 7.3 months); long delays occurred mostly during the start of the study.

**Table 2:** Data of the households (*n* = 22) assessed for *Salmonella* carriers.

**Index patient**					**Days between index case and household visit**	**Household member carriers of** ***Salmonella***			
**Household**	**Age/sex**	**Serotype**	**MLVA type**	**Date of sampling**		**Household identifier**	**Age/sex**	**Serotype**	**MLVA type**
HH1	18 months, F	Typhimurium	2-6-11-8-0210	26/05/2016	221	HH01	–	–	–
HH2	10 months, F	Enteritidis	2-13-3-3-NA	01/07/2016	208	HH02_11_II	7 years M	Typhimurium	2-7-10-8-0210
HH3	14 years, M	Typhi	–	28/09/2016	162	HH03	–	–	–
HH4	11 months, F	Typhimurium	2-5-8-8-0210	28/10/2016	131	HH04	–	–	–
HH5	5 years, M	Typhimurium	2-10-13-7-210	05/11/2016	123	HH05	–	–	–
HH6	5 months, M	Enteritidis	2-13-3-3-NA	07/11/2016	122	HH06_12	5 years F	II:42:r:-	–
**HH7**	**3.5 years, F**	**Enteritidis**	**2-13-4-3-NA**	**08/11/2016**	**61**	**HH07_23** ^*^	**3 years F**	**Enteritidis**	**2-13-4-3-NA**
						HH07_11	24 years M	Mikawasima	
HH8	4 years, M	Enteritidis	2-13-4-3-NA	15/11/2016	69	HH08_10	24 months M	Typhimurium	2-7-10-8-0210
HH9	10 months, M	Paratyphi C	–	06/06/2018	13	HH09	–	–	–
HH10	8 months, F	Enteritidis	2-13-4-3-NA	09/06/2018	5	HH10	–	–	–
HH11	11 months, M	Typhimurium	2-10-12-7-0210	02/07/2018	43	HH11	–	–	–
HH13	2.5 years, M	I 7 y:-	–	08/07/2018	11	HH13	–	–	–
HH14	6 years, M	Typhimurium	2-10-12-7-210	03/08/2018	32	HH14_02	11 years F	Typhimurium	2-5-9-8-0210
						HH14_03	11,5 years F	Typhimurium	2-5-9-8-0210
**HH15**	**15 months, F**	**Typhimurium**	**2-5-11-8-0210**	**07/08/2018**	**27**	**HH15_01** ^*^	**15 months F**	**Typhimurium**	**2-5-9-8-0210**
						**HH15_02**	**3 years F**	**Typhimurium**	**2-5-9-8-0210**
						**HH15_08**	**9 years F**	**Typhimurium**	**2-5-9-8-0210**
						**HH15_11**	**6 years F**	**Typhimurium**	**2-5-9-8-0210**
HH16	15 months, F	Enteritidis	2-13-4-3-NA	11/08/2018	23	HH16_01	15 months F	Typhimurium	2-5-9-8-0210
HH17	3 years, F	Typhi	–	09/12/2018	2	HH17	–	–	–
HH18	4 years, M	II:42:r:-	–	13/06/2019	20	HH18_11	7 years F	II:42:r:-	–
HH19	4 years, M	Typhimurium	2-NA-12-7-0210	14/06/2019	11	HH19	–	–	–
HH21	2.5 years, M	Enteritidis	2-14-4-3-NA	23/07/2019	4	HH21	–	–	–
HH22	19 months, M	Typhimurium	2-10-13-7-0210	24/12/2019	10	HH22	–	–	–
HH23	7 years, M	Typhi	–	15/01/2020	6	HH23_01	38 years F	Mikawasima	–
						HH23_05	9 years F	Mikawasima	–
HH24	9 months, M	Typhi	–	20/01/2020	12	HH24	–	–	–

Index patients with matching MLVA types among household members are indicated in bold. For index patients 5,568/4 and 6,284/4, the matching household member marked with ^*^ was the index patient who had recovered from bloodstream infection.

### Intestinal carriers among household members

Analysis of the first, second, and third stool samples from household members revealed five, nine, and two carriers, respectively. One carrier had *Salmonella* growth from two consecutive stool cultures, resulting in a total of 15 carriers. These 15 *Salmonella* carriers represented 7.3% of the 203 household members in nine of 22 (40.9%) households. Their median age was 7 years (15–38 years); 13 of 15 and five of 15 carriers were <15 and 5 years old, respectively; 14 (63.6%) were male individuals. The most prevalent serotype was *S*. Typhimurium (nine household members in five households). *Salmonella* serotypes Mikawasima, II:42:r:-, and Enteritidis were found in three, two, and one household members, respectively (Table 2).

### Index patients and corresponding household members with matching MLVA types

In two households, one from an index patient with *S*. Enteritidis and another from an index patient with *S*. Typhimurium, the MLVA types of the *Salmonella* from the index patient's blood culture matched the MLVA types of at least one stool sample in the corresponding household (Table 2, household numbers 7 and 15, respectively).

In the case of *S*. Enteritidis, the household member was the index case; the MLVA types of the blood and stool isolates were identical, and no other household members carried *Salmonella*. WGS showed that both the blood and stool isolates belonged to ST11 of the Central/Eastern African clade (HierBAPS clade 9, HC50_12675), as described by Feasey et al. (2016), and were closely related (three AD). Stool samples were obtained 61 days after the blood culture.

**Figure 1:**
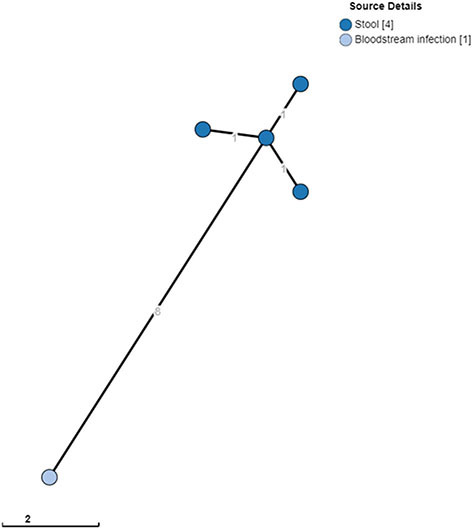
Clustering of *S*. Typhimurium ST313 from blood cultures and stool. A minimum spanning tree was created using the MSTree V2 component in EnteroBase based on the allelic differences over the 3,002 alleles that constitute the EnteroBase HierCC scheme on cgMLST (Zhou et al., 2021). The distances between leaves in the tree indicate the number of alleles different between genomes. Node colors are according to isolate origin.

In the case of *S*. Typhimurium, the index patient and three other household members carried *S*. Typhimurium. The isolates from the stool cultures shared the same MLVA type, which differed in one rapidly changing locus (i.e., STTR-6) from the MLVA type of the blood culture isolate. Stool samples were obtained 27 days after the blood culture. WGS revealed that the blood culture isolates, as well as the four stool culture isolates, belonged to ST313 lineage 2, as described by Pulford et al. (2021), and were closely related (0–1 AD among the stool isolates and eight AD with the blood culture isolate; Figure 1). All four *S*. Typhimurium carriers and one *S*. Enteritidis carrier were <10 years old.

The MLVA types of the two households with matching blood and stool culture isolates of *S*. Typhimurium and *S*. Enteritidis (household numbers 7 and 15, respectively) were also observed in other index patients and households (Table 2). *Salmonella* Typhimurium MLVA-type 2-5-9-8-210 from household 15 also occurred in households 14 and 16. The three households were sampled over 2 weeks but were located at a considerable distance (4.1 km) from each other. Similarly, *S*. Enteritidis MLVA type 2-13-4-3-NA also occurred in the index patients from households 8 and 16, sampled over 1 week.

Among the other non-typhoidal *Salmonella* serotypes, one index patient with *Salmonella* II:42:r:- was matched with a 7-year-old sibling from the corresponding household. In none of the four index patients with *Salmonella* Typhi, *Salmonella* was recovered from household members. In three households (household numbers 2, 8, and 16), the index patient was infected with *S*. Enteritidis, whereas the corresponding household member carried *S*. Typhimurium (Table 2).

### Antimicrobial resistance profiles

All *S*. Typhimurium (*n* = 9) and *S*. Enteritidis (*n* = 1) isolates from stool cultures were multidrug-resistant. This was in line with the results for the blood cultures of *S*. Typhimurium and *S*. Enteritidis isolates recovered during the study period [68.4% (13/19) and 76.9% (10/13) were multidrug resistant], but in contrast with the other *Salmonella* serotypes from stool cultures, which were all five pan-susceptible, i.e., susceptible to all antibiotics tested (Table 3).

**Table 3:** Antimicrobial resistance profiles of *Salmonella* serotype isolates recovered from index patients and their household members.

	**Index patients**				**Household members**		
	***Salmonella* Typhimurium (*n* = 19)**	***Salmonella* Enteritidis (*n* = 13)**	***Salmonella* Typhi + Paratyphi C (*n* = 8 + 1)**	**Other *Salmonella* (*n* = 2)**	***Salmonella* Typhimurium (*n* = 9)**	***Salmonella* Enteritidis (*n* = 1)**	**Other *Salmonella* (*n* = 5)**
Ampicillin	19 (100)	10 (76.9)	7 (77.7)	1	9 (100)	1	0
Trimethoprim-sulfamethoxazole	18 (94.7)	10 (76.9)	7 (77.7)	1	9 (100)	1	0
Chloramphenicol	13 (68.4)	10 (76.9)	6 (66.6)	0	9 (100)	1	0
Multidrug resistant (MDR)	13 (68.4)	10 (76.9)	5 (55.5)	0	9 (100)	1	0
Ceftriaxone	6 (31.5)	0	0	1	0	0	0
Azithromycin	2 (10.5)	0	0	1	0	0	0
Fluoroquinolone non-susceptible	6 (31.5)	0	6 (66.6)	1	0	0	0
MDR + fluoroquinolone non-susceptible	1 (5.2)	0	4 (44.4)	0	0	0	0

Data represent numbers (%) of intermediate susceptible and resistant isolates.

## Discussion

### Summary of findings

The present index patient-household member carriage study, conducted in an area endemic for iNTS infections, showed *S*. Typhimurium MLVA clusters in one household (including the infected index patient) and intestinal carriage of an identical *S*. Enteritidis MLVA type in the index patient of another household. Delays between blood and stool cultures were 27 and 61 days, respectively, and all carriers were <10 years of age. The matched blood and stool culture isolates belonged to the invasive ST313 lineage 2 (*S*. Typhimurium) and ST11 Central/Eastern African clade (*S*. Enteritidis).

### Limitations and strengths

The main limitations of this study are logistics and recruitment. In particular, at the start of the study, delays in communication of the microbiology report occurred, and the addresses of households in informal suburban settlements were unclear. In addition, the locations of the households were dispersed over a large area, precluding sensitization of the local community through communication with local leaders and health workers, as was done in a previous carrier study conducted in DR Congo (Mbuyi-Kalonji et al., 2020). The refusal ratio was also relatively high (*n* = 5 households), which may in part be related to the serious life-threatening condition of children, as observed previously in a hospital-based carrier study in DR Congo (Phoba et al., 2020).

Furthermore, because the research team comprised clinical and laboratory staff, preparing and mobilizing the teams for household visits took time. Consequently, only half of the eligible households were included, and the delays between blood culture sampling and household visits were twice as long as those in the aforementioned index patient-household study in Burkina Faso (median 13 days vs. 25 days in the present study) (Post et al., 2019). Furthermore, we did not include livestock in the present study. The main reason for this choice was the low number of livestock in the suburban slums, in contrast to a study in rural Burkina Faso (Post et al., 2019).

The strengths, sample transport, and laboratory work-up were validated (Post et al., 2019; Mbuyi-Kalonji et al., 2020) and managed for consistent quality. In addition, 3-day sampling (despite moderate participant compliance) substantially increased the cumulative proportion of carriers, as observed in a recent index patient-household study from Malawi (Koolman et al., 2022). As only 80.3 and 39.9% of the participants provided a second and third sample, respectively, it may be expected that the actual proportion of *Salmonella* carriers among household members would have been slightly higher than the actual 7.3%.

### Cumulative evidence of healthy human carriers as a potential reservoir of ints

The present study adds to the cumulative evidence that healthy human carriers are potential reservoirs of iNTS, particularly *S*. Typhimurium ST313. Previous index patient-household studies from Burkina Faso (rural areas) and Malawi (urban slums) also found matching index patient-household member pairs of *S*. Typhimurium ST313 and ST3257 (an ST type closely related to ST313), whereas animal and environmental sources did not reveal ST313 (Post et al., 2019; Koolman et al., 2022). For *S*. Enteritidis, so far no evidence of index patient-household control studies has been provided; this may partly be explained by the lower frequency of the *S*. Enteritidis serotype in the aforementioned studies.

Other evidence of healthy human carriers as potential reservoirs of iNTS was provided by a *Schistosoma*–*Salmonella* carrier study in a rural area in the Kongo Central province of DR Congo. In this study, four carriers of *S*. Typhimurium and *S*. Enteritidis had MLVA types similar to those of blood cultures at a nearby hospital (Mbuyi-Kalonji et al., 2020). Furthermore, healthy carriers of *Salmonella* Typhimurium ST313 were also reported in a large case-control diarrhea study in sub-Saharan Africa (Kasumba et al., 2021) and in blood–stool culture case-control studies in informal urban settlements in Kenya (both *S*. Typhimurium ST313 and *S*. Enteritidis ST11) (Kariuki et al., 2019, 2020). Paired blood–stool isolates of *S*. Typhimurium ST313 have been reported in 13 patients from the Central African Republic, but detailed information is lacking (Breurec et al., 2019).

### Long delay between stool and blood culture isolates in the index patients

In the present study, stool cultures were positive in two index patients infected with iNTS: *S*. Typhimurium and *S*. Enteritidis. The long delay between stool and blood cultures (27 and 61 days, respectively) supports the hypothesis of long-term carriage after systemic iNTS infection. A similar finding was observed in a hospital-based carrier study in the Kongo Central province of DR Congo (Phoba et al., 2020): stool cultures were performed in 299 children admitted with iNTS bloodstream infection; in nearly 30% of them, paired blood-stool isolates for *S*. Typhimurium ST313 and *S*. Enteritidis ST11 were found, of which two ST313 pairs with identical MLVA types had delays of 16 and 43 days, respectively (Phoba et al., 2020).

However, the above observations were anecdotical. Moreover, both studies were retrospective and provided information only at a single time point. Furthermore, given the long lag time between the index patient's blood cultures and household visits in the present study, the frequency of early convalescent shedding may have been missed. In the case of the household of the *S*. Typhimurium-infected index patients, stool samples, including that of the index patient, differed by eight AD from the blood culture sample, whereas the four stool cultures differed from each other by only one AD (Figure 1). This could raise the hypothesis that the index patient's isolate evolved slightly during the 27-day interval or may have evolved during exchanges between household members. Finally, the possibility of re-infection from a common source within a household cannot be excluded.

The potentially long duration of iNTS carriage contrasts with the short duration of fecal shedding demonstrated for diarrhea-causing non-typhoidal *Salmonella* (Gal-Mor, 2019) and raises the possibility of a “typhoid fever scenario,” with silent chronic iNTS carriers as reservoirs and sources of transmission (Kariuki et al., 2020; Phoba et al., 2020). However, this hypothesis should be further explored in longitudinal studies assessing carriage duration (Phoba et al., 2020). Furthermore, the incremental evidence of a human reservoir of the iNTS (Kariuki et al., 2019, 2020; Post et al., 2019; Kasumba et al., 2021; Koolman et al., 2022) and the absence of evidence for a major environmental reservoir (Crump et al., 2015, 2021) do not preclude co-existent environmental reservoirs and transmission routes of the iNTS (Kariuki et al., 2019; Mbae et al., 2020; Tack et al., 2021; Falay et al., 2022).

## Future research

Longitudinal cohort studies should assess the duration of iNTS carriage, patient age, and associated factors. *Salmonella* Typhimurium carriers in the present study were all children aged <15 years, and two household clusters of *S*. Typhimurium were noted. In the Burkina Faso study, an adult female household member carried ST313, and the other two index patients were siblings. Furthermore, household clusters (including clusters comprising human, livestock, and environmental isolates) of non-invasive non-typhoidal serotypes have been observed in Burkina Faso and Malawi studies (Post et al., 2019; Koolman et al., 2022), and this clustering could be addressed in future community-based studies.

In the present study, six of 22 households contained iNTS carriers, and the 10 iNTS carriers represented 4.9% of household members, with *S*. Typhimurium outnumbering *S*. Enteritidis (nine vs. one carrier). As no negative control households (i.e., households without index patients) were enrolled, we could not provide evidence of iNTS frequency among the entire population. Therefore, cross-sectional studies are required to further assess the population-based frequency of iNTS carriers. Finally, the retrospective study design did not allow for transmission assessment. Prospective, cohort-based field studies can provide such information but require a demographic health surveillance system and accessible microbiological diagnosis across the study area.

Regarding antimicrobial resistance, it is striking that MDR was confined to iNTS, which is a well-known phenomenon in sub-Saharan Africa and DR Congo (Tack et al., 2020a,b), whereas isolates belonging to the other serotypes were mostly pan-susceptible. This observation was previously made during a rat carrier study in Kisangani and may indicate distinct exposure to antibiotics among the iNTS and zoonotic *Salmonella* clades (Falay et al., 2022). To date, pan-susceptible *S*. Typhimurium ST313 lineage 3, which emerged in Malawi in 2016 (Pulford et al., 2021), has not been detected in DR Congo.

## Conclusion

The present study adds to the evidence of human carriers as reservoirs of the invasive *Salmonella* Typhimurium ST313 lineage 2 and *S*. Enteritidis ST11 Central/Eastern African clade. It demonstrated the household clustering of *S*. Typhimurium and the intestinal carriage of *S*. Typhimurium and *S*. Enteritidis in index patients several weeks after bloodstream infection.

## Data availability statement

Due to the sensitive nature of the data, the authors are unable to share the data directly. Requests to access the data can be made to ITM's contact point for data access (ITMresearchdataaccess@itg.be). All whole genome data are available at the ENA repository under project number PRJEB63268.

## Ethics statement

The studies involving humans were approved by Comité d'Éthique de l'École de Santé Publique de Université de Kinshasa Institutional review board of the ITM. The studies were conducted in accordance with the local legislation and institutional requirements. Written informed consent for participation in this study was provided by the participants' legal guardians/next of kin. Written informed consent was obtained from the individual(s), and minor(s)' legal guardian/next of kin, for the publication of any potentially identifiable images or data included in this article.

## Author contributions

Conceptualization: DF, DN, and JJ. Data curation: DF, LH, EB, and JJ. Formal analysis, investigation, and visualization: DF, LH, WM, and JJ. Funding acquisition: DN and JJ. Project administration: DF and JJ. Supervision: LH, OL, and DN. Writing—original draft: DF, LH, and JJ. Writing—review and editing: OL, EB, WM, and DN. All authors contributed to the article and approved the submitted version.

## Footnotes

^1^https://enterobase.warwick.ac.uk/ (accessed March 21, 2023).
